# Role of soil nutrient elements transport on *Camellia oleifera* yield under different soil types

**DOI:** 10.1186/s12870-023-04352-2

**Published:** 2023-08-02

**Authors:** Yu Chen, Jinjia Zheng, Zhijian Yang, Chenhao Xu, Penghui Liao, Shaosheng Pu, Yousry A. El-Kassaby, Jinling Feng

**Affiliations:** 1 College of Forestry, Fujian Agriculture and Forestry University, Fuzhou, 350002 China; 2 Popularization Station of Forestry Science Technology of Fujian Province, Fuzhou, 350003 Fujian China; 3 Department of Forest and Conservation Sciences, Faculty of Forestry, University of British Columbia, 2424 Main Mall, Vancouver, BC V6T 1Z4 Canada

**Keywords:** Soil conditions, Key nutrient elements, Organ, Yield regulation, Oil composition regulation, *Camellia oleifera*

## Abstract

**Background:**

Most of *Camellia oleifera* forests have low fruit yield and poor oil quality that are largely associated with soil fertility. Soil physical and chemical properties interact with each other affecting soil fertility and *C. oleifera* growing under different soil conditions produced different yield and oil composition. Three main soil types were studied, and redundancy, correlation, and double-screening stepwise regression analysis were used for exploring the relationships between *C. oleifera* nutrients uptake and soil physical and chemical properties, shedding light on the transport law of nutrient elements from root, leaves, and kernel, and affecting the regulation of fruit yield and oil composition.

**Results:**

In the present study, available soil elements content of *C. oleifera* forest were mainly regulated by water content, pH value, and total N, P and Fe contents. Seven elements (N, P, K, Mg, Cu, Mn and C) were key for kernel’s growth and development, with N, P, K, Cu and Mn contents determining 74.0% the yield traits. The transport characteristics of these nutrients from root, leaves to the kernel had synergistic and antagonistic effects. Increasing oil production and unsaturated fatty acid content can be accomplished in two ways: one through increasing N, P, Mg, and Zn contents of leaves by applying corresponding N, P, Mg, Zn foliar fertilizers, while the other through maintaining proper soil moisture content by applying Zn fertilizer in the surface layer and Mg and Ca fertilizer in deep gully.

**Conclusion:**

Soil type controlled nutrient absorption by soil pH, water content and total N, P and Fe content. There were synergistic and antagonistic effects on the inter-organ transport of nutrient elements, ultimately affecting N, P, K, Cu and Mn contents in kernel, which determined the yield and oil composition of *C. oleifera.*

## Introduction

*Camellia oleifera* belongs to the genus *Camellia* of Theaceae family, and is one of the four largest woody oil plants in the world [1]. At present, the planted area of *C. oleifera* in China is 4.5 × 10^6^ hm^2^, but most of its forests have low fruit yield and poor oil quality, restricting efficient industrial development [1, 2]. These limitations are largely associated with soil fertility [3]. Soil physical and chemical properties interact with each other to affect soil fertility. Thus, their appropriate properties not only improve nutrient elements availability, but also their absorption, transport, transformation, and assimilation by plants [4]. Nutrient elements availability is related to soil adsorption and fixation capacity and the degree of coordination and antagonism among elements, which in turn is affected by soil type and nutrient elements characteristics [5–7]. Recently, *C. oleifera* nutrient elements utilization and absorption research has been mostly focused on soil nutrient abundance/deficiency, with little attention to soil physical and chemical properties influences, nutrient adsorption characteristics, and soil element interactions [8]. Nutrient elements participate in a series of physiological and biochemical processes such as metabolism, energy transformation, and electron transport of plant organs, and represent the basic material for plant growth and development, and yield quality (e.g., oil content and composition) [9]. Different studies have concluded that the main soil factors are associated with soil total N, organic matter, and hydrolyzed N [10], or soil total K, organic matter, and available Fe [11], or soil N/P ratio [2, 12]. Therefore, it is essential to understand soil conditions, nutrient elements absorption capacity, and yield traits of *C. oleifera* forests to enhance their efficiency, economy, and sustainable development.

Plants growth and development are restricted by soil nutrient availability, and affected by differences in nutrient storage and various organs functional differentiation, resulting in different nutrient elements distribution within the same plant’s different organs [13]. This was observed in *C. oleifera* growing under different soil conditions which subsequently affected their fruit yield and oil composition [8, 11, 12]. Nutrient elements content in plant leaves and roots can characterize the demand and utilization of nutrients [5], while those in leaves and fruits can determine fruit yield and oil quality [14]. Recently, research studies have been focused on the dynamic changes and correlation of nutrient elements in *C. oleifera* leaves, fruits, and soil [8, 11, 12, 15]; however, these studies lacked information on nutrient elements distribution among different organs, and how they interact and transport from the soil to the kernel, information essential to the proper nutritional management of *C, oleifera*.

Soil role on absorption, transport, and utilization of plant nutrient elements is controlled by multiple factors. Current research only considers the quantitative relationship between a particular element within the soil or an organ and vice versa, but does not consider the internal comprehensive/holistic relationship among various group of elements, and does not clarify multiple elements relationship within and between the soil, or organs [14–16]. Redundancy analysis and double-screening stepwise regression analysis can be used to better analyze the relationship between variables within and between the soil, or organs [17–19]. Therefore, in order to deeply understand the relationship between *C. oleifera* yield and fruit traits with those of roots, leaves, kernels and soil, *C. oleifera* forests from different soil types (red, yellow-red. and purple soil) in Fujian Province, China were selected to address this issue. Here, the physical and chemical properties of these three soil types (red, yellow-red. and purple), contents of 11 nutrient elements in roots, leaves, and kernels, as well as morphological and yield traits of fruits were determined. Redundancy and correlation analyses and double-screening stepwise regression analysis were used to explore the: (1) correlation between the nutrient requirements of *C. oleifera* and soil mineral elements and physical properties; (2) transport law of nutrient elements from roots, leaves to kernels; and (3) mechanism regulating *C. oleifera* fruit and yield traits through forest soil type and leaves. Thus, the aim of this study is to clarify the effects of soil physical and chemical properties and plant nutrient elements on *C. oleifera* fruit yield and oil composition. The generated information is expected to provide valuable insights for understanding *C. oleifera* nutrient contents and fruit characteristics for high-yield and high-quality production, and to provide a theoretical basis and guidance for soil, fertilization, and fruit yield and oil quality improvement of *C. oleifera*.

## Results

### ***C. oleifera*** different forest types soil physical and chemical characteristics

Total porosity, non-capillary porosity, and total C content of the yellow-red, purple, and red soils in the 20 ~ 40 cm soil layers were all lower than those in the 0 ~ 20 cm soil layer (Table 1). However, the soil bulk density and soil water contents, pH value, total K, Mg, Ca, Fe, Cu, and Zn in the 0 ~ 20 cm soil layer were all greater than those in the 0 ~ 20 cm soil layer, indicating that the total porosity, non-capillary porosity and total C content of the soil decreased, while soil bulk density, soil water content, pH value, total K, Mg, Ca, Fe, Cu, and Zn contents increased with the three soil types depth. The capillary water holding capacity, soil saturated water content, total N and Mn contents of the yellow-red soil, capillary porosity, total P and Al contents of purple soil, total N and Mn contents of red soil were less than those of 0 ~ 20 cm soil, while soil other indices were greater than those of 0 ~ 20 cm soil. These indicated that moisture holding capacity, soil saturated water content, total N and Mn contents decreased with the depth of the yellow-red soil layer; capillary porosity, total P and Al contents decreased with the depth of the purple soil layer; and total N and Mn contents decreased with the depth of the red soil layer; however, these indexes in the other soil types increased with soil layer depth.

**Table 1:** Physical and chemical properties of *C. oleifera* three forest soil types

Indicators	0 ~ 20 cm				20 ~ 40 cm		
	Yellow-red soil	Purple soil	Red soil		Yellow-red soil	Purple soil	Red soil
Soil bulk density (g·cm^− 3^)	1.03 ± 0.07b	1.03 ± 0.07b	1.24 ± 0.03a		1.33 ± 0.10b	1.26 ± 0.02b	1.45 ± 0.07a
Total soil porosity %	0.48 ± 0.01b	0.44 ± 0.01c	0.50 ± 0.02a		0.45 ± 0.01b	0.40 ± 0.01c	0.48 ± 0.01a
Capillary porosity %	0.34 ± 0.04c	0.40 ± 0.01b	0.45 ± 0.02a		0.41 ± 0.02b	0.37 ± 0.01c	0.46 ± 0.01a
Non-capillary porosity %	0.14 ± 0.03a	0.03 ± 0.01b	0.05 ± 0.00b		0.04 ± 0.01a	0.03 ± 0.00b	0.02 ± 0.01c
Soil moisture content %	0.17 ± 0.01b	0.11 ± 0.02c	0.26 ± 0.02a		0.20 ± 0.01b	0.13 ± 0.03c	0.30 ± 0.01a
Soil moisture holding capacity %	0.36 ± 0.01b	0.34 ± 0.01c	0.41 ± 0.01a		0.32 ± 0.01c	0.37 ± 0.01b	0.47 ± 0.02a
Soil saturated water content %	0.49 ± 0.02a	0.33 ± 0.02c	0.44 ± 0.01b		0.37 ± 0.03b	0.36 ± 0.03b	0.47 ± 0.01a
Soil pH	4.73 ± 0.09b	5.59 ± 0.28a	4.64 ± 0.04b		4.88 ± 0.07b	6.76 ± 0.23a	4.67 ± 0.04c
Total C content (g·Kg^− 1^)	21.31 ± 2.18a	1.86 ± 0.16b	2.73 ± 0.38b		6.68 ± 1.21a	1.51 ± 0.19c	2.56 ± 0.25b
Total N content (g·Kg^− 1^)	1.09 ± 0.13a	0.15 ± 0.04c	0.44 ± 0.06b		0.64 ± 0.07a	0.23 ± 0.02c	0.32 ± 0.06b
Total P content (g·Kg^− 1^)	0.35 ± 0.02b	0.24 ± 0.03c	0.48 ± 0.05a		0.61 ± 0.08a	0.18 ± 0.02c	0.55 ± 0.06b
Total K content (g·Kg^− 1^)	17.36 ± 0.86a	14.79 ± 0.92b	11.47 ± 1.57c		22.80 ± 3.14a	18.09 ± 0.92b	12.38 ± 1.35c
Total Mg content (g·Kg^− 1^)	4.52 ± 0.36b	3.90 ± 0.43b	7.35 ± 0.95a		5.17 ± 0.61b	4.01 ± 0.63c	7.83 ± 1.71a
Total Ca content (g·Kg^− 1^)	4.50 ± 0.24a	3.18 ± 0.26c	3.86 ± 0.38b		5.02 ± 0.53a	3.45 ± 0.26c	4.32 ± 0.41b
Total Al content (g·Kg^− 1^)	457.61 ± 39.76a	260.40 ± 39.34c	360.78 ± 31.54b		543.15 ± 20.90a	236.47 ± 30.12c	480.07 ± 52.08b
Total Mn content (g·Kg^− 1^)	0.45 ± 0.03c	1.64 ± 0.19a	1.03 ± 0.06b		0.37 ± 0.02c	2.28 ± 0.29a	0.61 ± 0.05b
Total Fe content (g·Kg^− 1^)	90.12 ± 4.15c	110.82 ± 7.40b	191.42 ± 16.13a		109.41 ± 7.58b	110.17 ± 9.63b	200.17 ± 14.27a
Total Cu content (mg·kg^− 1^)	0.54 ± 0.02a	0.44 ± 0.03b	0.34 ± 0.02c		0.60 ± 0.02a	0.47 ± 0.03b	0.38 ± 0.02c
Total Zn content (mg·kg^− 1^)	0.12 ± 0.02b	0.16 ± 0.01a	0.09 ± 0.01c		0.13 ± 0.01b	0.18 ± 0.01a	0.10 ± 0.01c

Means within the same line with different letters indicate significant differences (*P* < 0.05). Soil total N content less than 0.75 g·Kg^− 1^ was at the very low level, and between 0.75 and 1.00 g·Kg^− 1^ was at the low level; soil total P content less than 0.90 g·Kg^− 1^ was at the very low level; soil total K content with 9.00 ~ 12.00 g·Kg^− 1^ was at a low level, 12.00 ~ 18.00 g·Kg^− 1^ was at a medium level, and 18.00 ~ 25.00 g·Kg^− 1^ was at a high level [20]. Average soil total Mg content in southern China was 5 g·Kg^− 1^ [21]. Average soil total Ca content was 36.4 g·Kg^− 1^ [22]; average soil total Mn content was 0.40 g·Kg^− 1^; average soil total Al content was 68.90 g·Kg^− 1^; average soil total Fe content was 50 g·Kg^− 1^ [18]. Average soil total Cu content in soil was 0.40 g·Kg^− 1^ [23]. Average of soil total Zn content was 0.05 g·Kg^− 1^ [24]

### Effects of different soil types on the element contents in various ***C. oleifera*** organs

**Fig. 1:**
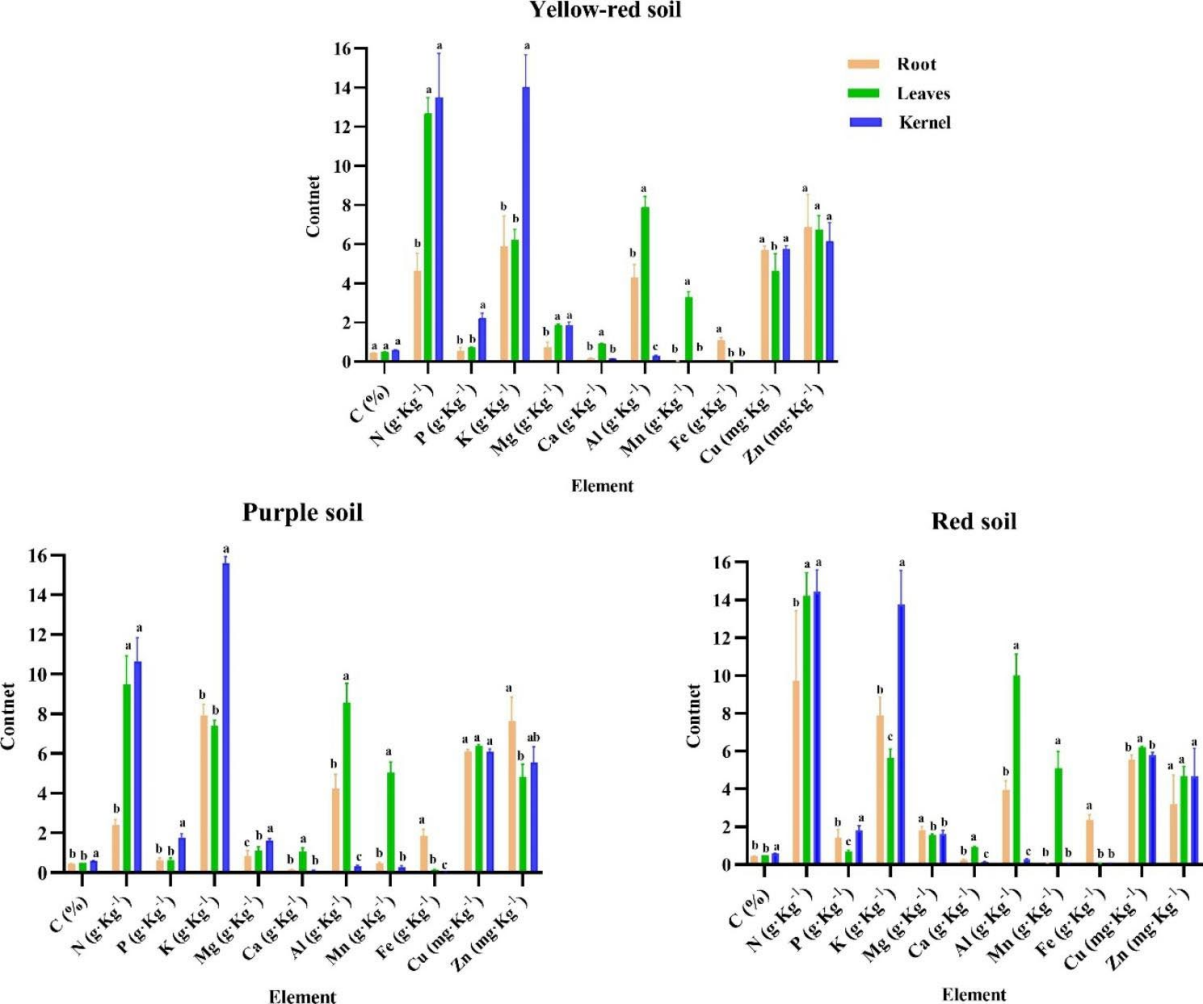
Effects of three soil types on the element contents of *C. oleifera* root, leaves and kernel. Different letters indicate significant differences in root, leaves and kernel of *C. oleifera* (*P* < 0.05)

Significant differences were observed in the element contents in various *C. oleifera* organs across the three different soil types (Fig. 1). In the yellow red soil, root Zn content, leaves total C, Mg, Ca and Zn contents, and kernel total C, N, P, K, and Mg contents were the highest. In the purple soil, root total Fe, Cu and Zn contents, leaves total Ca, Al, Mn and Cu contents, and kernel total C, N, P, K, Mg and Mn contents were the highest. In red soil, root total Mg and Fe contents, leaves total N, Al, Mn and Cu contents, and kernel total C, N, P, K and Zn contents were the highest. Arranged according to the value of each element content, kernels were C > K > N > P > Mg > Al > Ca > Mn > Fe > Cu > Zn; leaves were C > N > Al > K > Mn > Mg > Ca > P > Fe > Zn > Cu; and roots were C > K > N > Al > Mg > P > Ca > Mn > Fe > Zn > Cu (Fig. 1). In addition to Cu and Zn content, the contents of the other nine elements were significantly different in root, leaves and kernel. Fe content was the highest in root, Ca, Al and Mn in leaves, C, P and K in kernel, N in leaves and kernel, indicating that *C. oleifera* root was the enrichment organ of Fe, leaves was the enrichment organ of Ca, Al, Mn and N, and kernel was the enrichment organ of C, N, P and K.

### ***C. oleifera*** fruit characteristics and yield components under different soil types

**Fig. 2:**
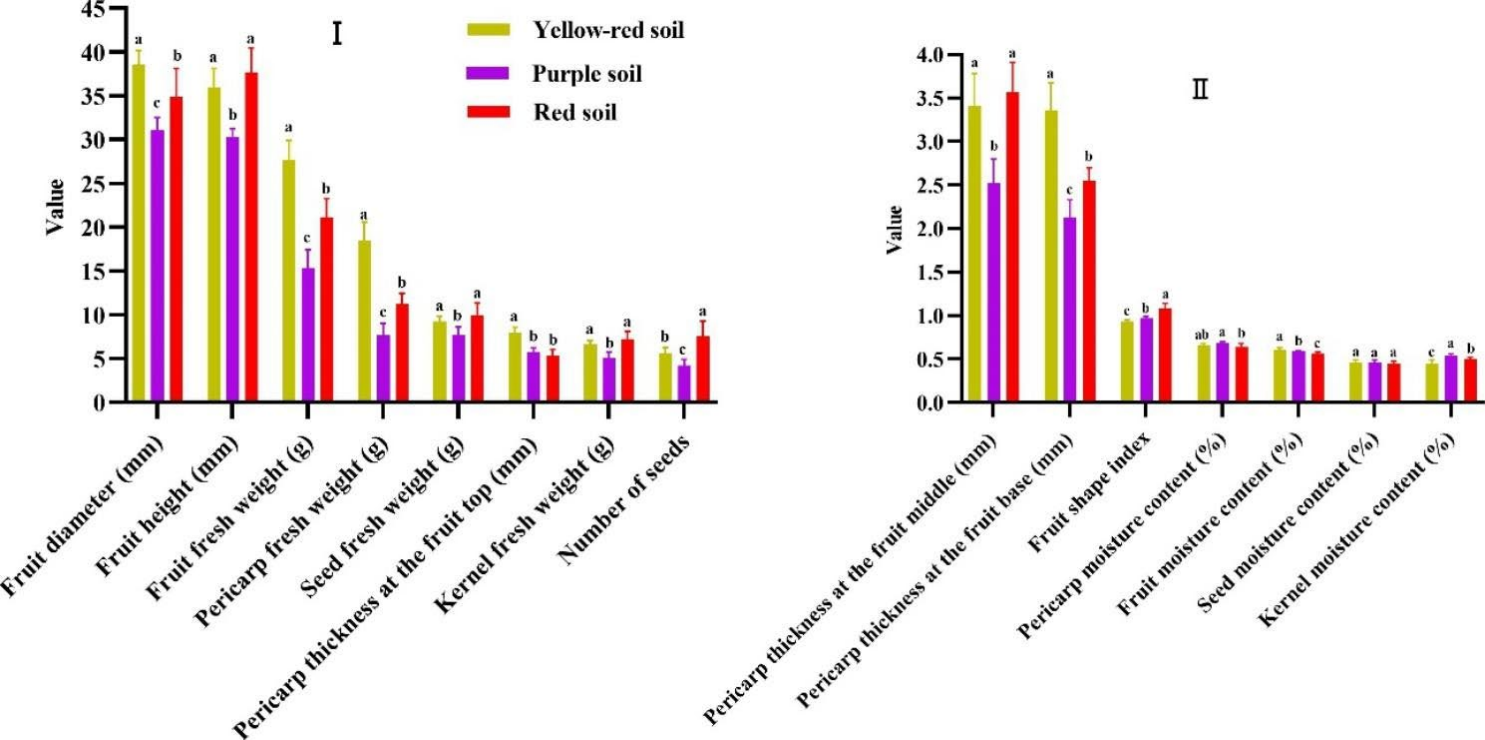
Effects of three soil types on fruit characters of *C. oleifera.* Different letters indicate significant differences in three soil types (*P* < 0.05)

With the exception of seed moisture content, significant differences between the different soil types were observed for fruit characters (Fig. 2). Fruit diameter, fruit fresh weight, pericarp fresh weight, pericarp thickness at the fruit top, thickness at the fruit middle, thickness at the fruit base, fruit and seed moisture contents were the largest in the yellow red soil. Pericarp moisture content and Kernel moisture content were the largest in the purple soil. Fruit height, seed fresh weight, kernel fresh weights, number of seeds, pericarp thickness at the fruit middle, and fruit shape index were the highest in the red soil.

Significant differences in oil yield traits were observed among the different soil types (Table 2). Fruit yield, dry kernel oil content, oil production and unsaturated fatty acid content were the highest in yellow-red soil. Palmitic and linoleic acid contents, other fatty acid contents, saturated fatty acid content and saturated/unsaturated ratio were the highest in purple soil. Oil content of fresh fruit, stearic, oleic and linolenic acid contents were the highest in red soil.

**Table 2:** Effects of different soil types on *C. oleifera* yield traits

Indicators	Yellow-red soil	Purple soil	Red soil
The fruit yield (g·plant^− 1^)	5918.89 ± 224.86a	5201.11 ± 759.89b	4036.67 ± 436.92c
Dry kernel oil content %	41.23 ± 0.88a	30.47 ± 0.94c	36.60 ± 0.66b
Oil content of fresh fruit%	5.45 ± 0.82b	4.63 ± 0.39c	6.28 ± 0.49a
Oil production (g·plant^− 1^)	322.79 ± 30.23a	230.00 ± 31.71b	253.50 ± 35.25b
Palmitic acid content %	9.01 ± 0.06b	9.59 ± 0.04a	9.06 ± 0.07b
Stearic acid content %	1.69 ± 0.09c	1.85 ± 0.08b	2.06 ± 0.06a
Oleic acid content %	80.95 ± 0.17a	78.62 ± 0.57b	81.22 ± 0.15a
Linoleic acid content %	6.60 ± 0.07b	7.38 ± 0.22a	5.39 ± 0.10c
Linolenic acid content %	0.25 ± 0.01b	0.16 ± 0.00c	0.35 ± 0.04a
Other content %	1.50 ± 0.18c	2.40 ± 0.38a	1.90 ± 0.10b
Saturated fatty acid content %	10.70 ± 0.09c	11.44 ± 0.43a	11.13 ± 0.08b
Unsaturated fatty acid content %	87.81 ± 0.17a	86.16 ± 0.60c	86.97 ± 0.13b
Saturated/unsaturated ratio	0.122 ± 0.001c	0.133 ± 0.006a	0.128 ± 0.001b

Means within the same line with different letters indicate significant differences (*P* < 0.05)

### Relationship between fruit and yield traits and leaves elements content of ***C. oleifera***

**Fig. 3:**
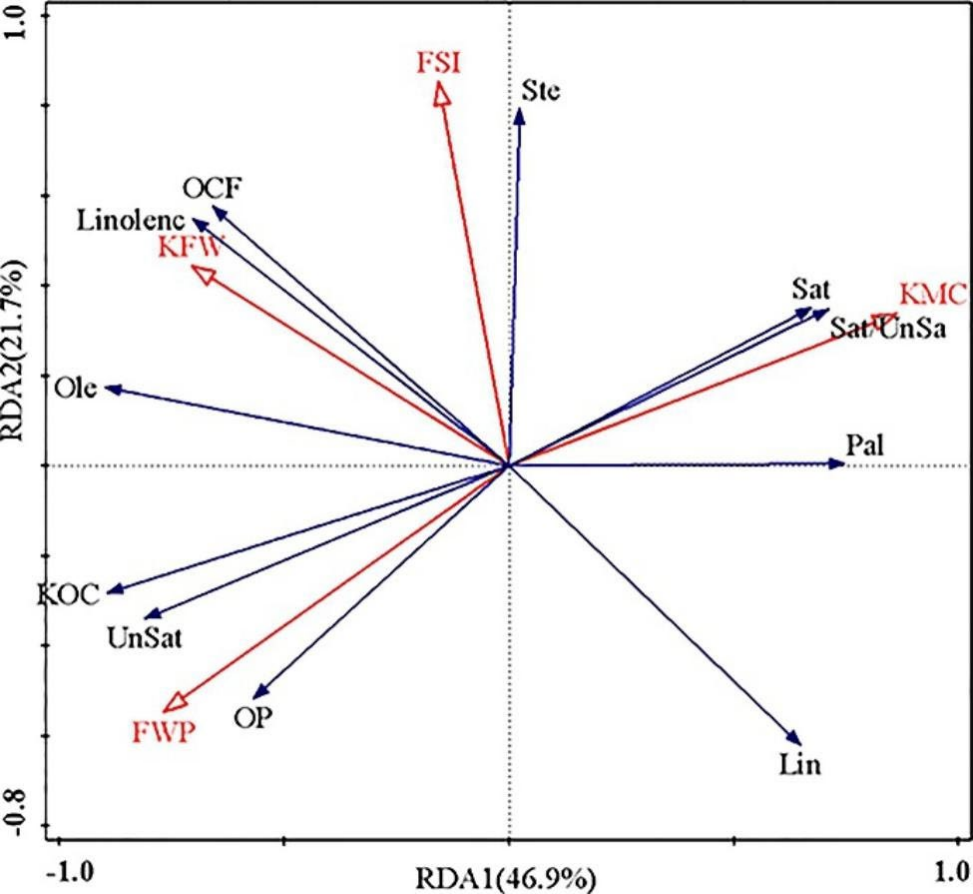
Redundancy analysis of the relationship between *C. oleifera* fruit and fruit production traits

By selecting fruit morphological indices as explanatory variables and yield traits as response variables, a two-dimensional ranking map of yield traits and fruit morphological indices was obtained (Fig. 3). The interpretation rate of the first and second axes of the redundancy analysis (RDA) were 46.9 and 21.7%, respectively, for a total interpretation rate of 68.6%. Fruit shape index (FSI), kernel moisture content (KMC), fresh weight of pericarp (FWP) and kernel fresh weight (KFW), all contributed to the main effect on yield traits. According to the vector angle analysis, FWP is positively and significantly correlated with oil production (OP), unsaturated fatty acid content (Unsat) and dry kernel oil content (KOC). KMC is positively and significantly correlated with palmitic acid content (Pal), saturated fatty acid content (Sat) and saturated/unsaturated ratio (Sat/ Unsat). KFW and FSI are positively and significantly correlated with oil content of fresh fruit (OCF), linolenic acid content (Linolenc), oleic acid content (Ole) and stearic acid content (Ste). OP was positively and highly significantly correlated with Unsat, KOC, and Ole, and negatively and highly significantly correlated with Pal, Sat/ Unsat, Sat and Ste.

**Fig. 4:**
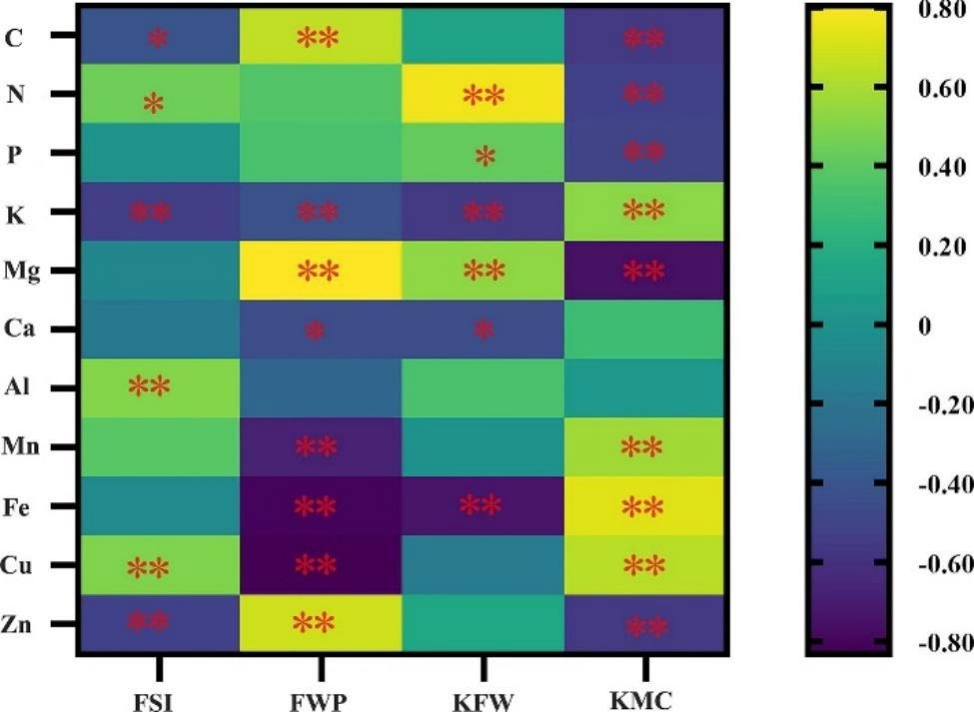
Correlation analysis of *C. oleifera* main fruit characters and leaves element contents. * and ** indicate significant and highly significant correlation

Further, the correlation analysis between fruit main traits and leaves elements contents (Fig. 4), indicated that FSI is negatively and significantly correlated with leaves C content, negatively and highly significantly correlated with leaves K and Zn content. FSI is positively and significantly correlated with leaves N content, and positively and highly significantly correlated with leaves Al and Cu content. FWP is positively and highly significantly correlated with leaves C, Mg and Zn contents. FWP is negatively and significantly correlated with leaves K and Ca content, and negatively and highly significantly correlated with leaves Mn, Fe and Cu content. KFW was positively and highly significantly correlated with leaves N and Mg content, positively and significantly correlated with leaves P content. KFW is negatively and highly significantly correlated with leaves K and Fe content, and negatively and significantly correlated with leaves Ca content. KMC is negatively and highly significantly correlated with leaves C, N, P, Mg and Zn contents. KMC is positively and highly significantly correlated with the contents of leaves K, Mn, Fe and Cu content.

### Soil, organs and yield elements contents interactions

**Fig. 5:**
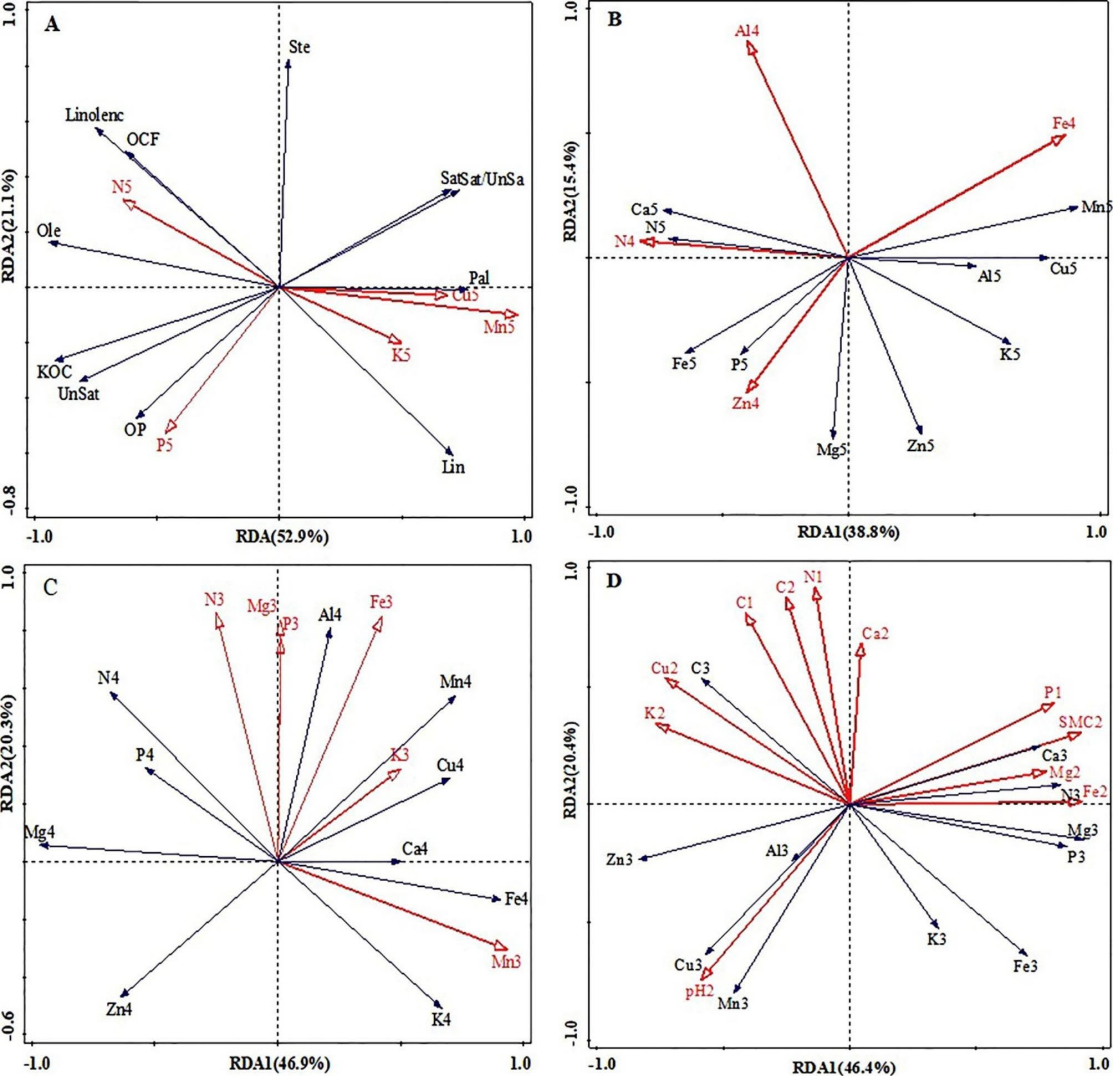
Redundancy analysis of elements contents among soil, plant organs, and yield. The numbers (1, 2, 3, 4 and 5) following each character correspond to: (1) 0 ~ 20 cm soil layer. (2) 20 ~ 40 cm soil layer, (3) root element,4) leaf element, and 5) kernel element, respectively

Figure 5-A shows the two-dimensional ranking diagram of *C. oleifera* kernel elements contents (as the explanatory variables) and yield traits (as the response variables). The interpretation rate of the first and second axes of RDA are 52.9 and 21.1%, respectively, with a total interpretation rate of 74.0%. Kernel P, N, Cu, Mn, and K content contributed to the main effects on yield traits, with P > Mn > Cu > N > K. By comparing the vertical projection length, kernel total P content had the largest positive effect on OP, Unsat and KOC; kernel N content had the largest positive effect on ole and Linolenc, OCF and Ste. According to the vector angle analysis, kernel K, Cu and Mn content were negatively and highly significantly correlated with OP, and positively and highly significantly correlated with Sat and Pal and Lin contents, kernel total N and P content were positively significantly correlated with Linolenc, OCF, Ole, KOC, Unsat and OP, negatively significantly correlated with Sat, Sat/UnSa, and Pal.

Figure 5-B shows the two-dimensional ranking diagram of *C. oleifera* kernel elements contents (as the explanatory variables) and leaves elements contents (as the response variables). The interpretation rate of the first and second axes of RDA are 38.8 and 15.4%, respectively, with a total interpretation rate of 54.2% with leaves total Al, Fe, Zn and N contents contributing to the main effect on kernel elements contents. By comparing the vertical projection length, leaves total Zn content had the largest positive effect on kernel total P and Mg content; leaves total N content had the largest positive effect on kernel total N content, and had the largest negative effect on kernel total Cu and Mn contents; leaves total Fe content had the greatest positive effect on kernel total Mn content; leaves total Al and N contents had the largest negative effect on kernel total K content. According to the vector angle analysis, there were no significant relationships between kernel total P, Mn, N, K, Mg contents and those in the leaves.

Figure 5-C shows the two-dimensional ranking diagram of *C. oleifera* leaves elements contents (as the response variables) and root elements contents (as the explanatory variables). The interpretation rate of the first and second axes of RDA are 46.9 and 20.3%, respectively, with a total interpretation rate of 67.2%. Root total N, Mg, P, Fe, K and Mn contents contributing to the main effect on leaves elements contents. By comparing the vertical projection length, root total K content had the greatest negative effect on leaves total Zn content; root total N content had the greatest positive effect on leaves total N and P contents; root total Mn content had the greatest positive effect on leaves total Fe content; root total Mg, P and Fe content had the greatest positive effect on leaves total Al content; root total Mn content had the greatest positive effect on leaves total K content; root total N content had the largest negative effect on leaves total K content and the largest positive effect on leaves total Mg content. Through vector angle analysis, leaves total P, Mn, N and K contents were very positively and significantly correlated with root total P, Mn, N and K contents; leaves total Cu and Mn contents were very positively and significantly correlated with root total K content; and leaves total Mg content was not significantly correlated with root total Mg content.

Figure 5-D shows the two-dimensional ranking diagram of *C. oleifera* root elements contents (as the response variables) and soil physical and chemical properties (as the explanatory variables). The interpretation rate of the first and second axes of RDA are 46.4 and 20.4%, respectively, with a total interpretation rate of 66.8%. The contents of total P, C and N in 0 ~ 20 cm soil layer, and pH, the contents of total K, Cu, C, Ca, Mg, Fe and SMC in 20 ~ 40 cm soil layer had the main effects on root elements contents. By comparing the vertical projection length, SMC in 20 ~ 40 cm soil layer had the greatest positive effect on root total N, Mg and P contents, had the greatest negative effect on root total Zn content; total C content in 0 ~ 40 cm soil layer and the total N of 0 ~ 20 cm soil layer had the greatest negative effect on root total Fe and K content; the pH of 20 ~ 40 cm soil layer had the greatest effect on root total Cu, Mn and Zn contents. According to the vector angle analysis, root total N content was significantly and negatively correlated with the total N content in 0 ~ 20 cm soil layer. Root P content was highly significantly and positively correlated with the total P content in 0 ~ 20 cm soil layer. Root total K content was highly significantly and negatively correlated with the total K content in the 20 ~ 40 cm soil layer, and significantly and positively correlated with pH value. Root total Mg, Fe, Cu and Ca contents were significantly and positively correlated with total Mg, Fe, Cu and Ca content in 20 ~ 40 cm soil layer, indicating that *C. oleifera* root N, P, K, Mg, Fe, Cu and Ca absorption were significantly dependent on the total amount of these elements in the soil. Root Mg, Fe and Ca content were significantly positively affected, and Cu content negatively by SMC. Root total Mn, Al and Zn contents were not significantly correlated with total Mn, Al and Zn content in soil, but were positively and significantly correlated with soil pH, and soil pH significantly and negatively correlated with SMC in 20 ~ 40 cm soil layer, indicating that root Mn, Al and Zn absorption had no relationship with these elements total amount in the soil, and that the absorption was determined by SMC. Soil total Mg, Fe, and P contents, and SMC in the 20 ~ 40 cm soil layers were positively and significantly correlated with root N, P, Mg and Ca contents, negatively and significantly correlated with the pH in 20 ~ 40 cm soil, and root Al, Cu, Mn and Zn contents. Total Ca, N and Cu contents in the 20 ~ 40 cm soil layers were significant and negatively correlated with root K content. SMC and total Fe content in the 20 ~ 40 cm soil layer had the greatest effect on root P content.

### ***C. oleifera*** double-screening stepwise regression analysis between yield traits and soil physicochemical properties

Here, the 20-soil physical and chemical indices in the 0 ~ 20 cm and 20 ~ 40 cm soil layers were used as independent variables, and the 12-yield traits were used as dependent variables to carry out the double-screening stepwise regression analysis, and the regression equations for the main yield traits were established with being highly significant (Table 3). For the 0 ~ 20 cm soil layer, the regression equations indicated that soil attributes (SMC, soil moisture holding capacity, saturated water content, pH, total C, N, K, Mg, and Zn contents) were significantly correlated with yield traits (KOC, OP, Ole, Lin, and Unsat), indicating that the 0 ~ 20 cm soil layer determined *C. oleifera* KOC, OP, Ole, Lin, Linolenc and Unsat (Table 3). The coefficients of independent variables in each regression equation, indicated that soil moisture holding capacity had the largest significant positive effect, soil total Zn content had the largest significant negative effect on KOC; soil total Zn content had the largest significant positive effect on OP, while soil moisture holding capacity had the largest significant negative effect on OP; SMC had the largest positive effect on Ole, while pH had a negative effect on Ole; soil total Zn content had the largest significant positive effect on Lin, and soil moisture holding capacity had the largest significant negative effect on Lin; soil total Zn content had the largest positive effect on Linolenc, while soil total C content had a negative effect on Linolenic; soil Zn content had the most significant positive effect on Unsat, while soil moisture holding capacity had a significant negative effect on Unsat.

For the 20 ~ 40 cm soil layer, the regression equations indicated that soil attributes (soil saturated water content, pH, total C, Mg, Ca, Mn and Fe contents) were significantly correlated with yield traits (the fruit yield, KOC, OP, Ste and Lin), indicating that the 20 ~ 40 cm soil layer determined *C. oleifera* fruit yield, KOC, OP, stearic acid and Lin (Table 3). The coefficients of independent variables in each regression equation, indicated that the total Mg content had the largest significant positive effect on the fruit yield, while soil saturated water content had the largest significant negative effect on the fruit yield; soil total Ca content had the largest significant positive effect on KOC, and soil pH had the largest significant negative effect on KOC; soil saturated water content had the largest significant positive effect on OP, and soil total Mn content had the largest significant negative effect on OP; soil saturated water content had a significant maximum positive effect on Ste, and soil total Mn content had a significant maximum negative effect on Ste; soil pH had the largest significant positive effect on Lin, and soil saturated water content had the largest significant negative effect on Lin.

**Table 3:** Double-screening between *C. oleifera* soil physicochemical properties and yield traits and the development of stepwise regression equations

Soil layer	Double-screening stepwise regression equation	Correlation coefficient	R^2^	*F* value
0 ~ 20 cm	Y_2_ = 40.31 + 23.88 × _6_ + 14.57 × _7_ -1.33 × _8_ + 0.329 × _9_ -0.480 × _12_ -31.589 × _20_	0.989	0.979	125.890**
	Y_4_ = -92.56 -200.60 × _5_ -796.66 × _6_ + 51.43 × _8_ -1.6906 × _9_ + 168.52 × _10_ + 40.20 × _14_ + 609.91 × _20_	0.943	0.889	18.045**
	Y_7_ = 0.778 + 0.091 × _5_ + 0.017 × _6_ -0.006 × _8_ + 0.001 × _9_ + 0.001 × _10_ + 0.002 × _14_ + 0.07 × _20_	0.967	0.934	31.932**
	Y_8_ = 0.034 -0.054 × _6_ + 0.034 × _7_ + 0.008 × _8_ -0.001 × _9_ + 0.092 × _20_	0.989	0.978	122.340**
	Y_9_ = -0.009 + 0.005 × _5_ + 0.015 × _6_ + 0.001 × _8_ -0.001 × _9_ + 0.003 × _10_ + 0.001 × _14_ + 0.020 × _20_	0.944	0.891	18.300**
	Y_11_ = 0.802 + 0.057 × _5_ -0.017 × _6_ + 0.003 × _8_ + 0.009 × _10_ + 0.002 × _14_ + 0.144 × _20_	0.921	0.847	12.491**
20 ~ 40 cm	Y_1_ = 9802.91 -10422.579 × _7_ + 225.129 × _8_ + 48.32 × _9_ + 290.99 × _14_ -218.61 × _15_ -494.899 × _17_ -15.43 × _18_	0.945	0.892	22.488**
	Y_2_ = 45.21 -0.03 × _7_ -2.43 × _8_ + 0.578 × _9_ -0.325 × _14_ + 1.376 × _15_ -0.754 × _17_ -0.008 × _18_	0.992	0.983	157.042**
	Y_4_ = 495.56 + 94.49 × _7_ -14.94 × _8_ + 3.77 × _9_ + 16.66 × _14_ -18.62 × _15_ -28.44 × _17_ -1.29 × _18_	0.909	0.871	12.981**
	Y_6_ = 0.011 + 0.011 × _7_ + 0.001 × _8_ -0.0006 × _9_ + 0.001 × _14_ + 0.001 × _15_ -0.0013 × _17_	0.914	0.859	13.830**
	Y_8_ = 0.051 -0.013 × _7_ + 0.004 × _8_ + 0.001 × _9_ -0.001 × _14_ + 0.001 × _15_ + 0.002 × _17_ -0.001 × _18_	0.989	0.978	122.077**

Y_1_: The fruit yield; Y_2_: KOC; Y_4_: OP; Y_6_: Ste; Y_7_: Ole; Y_8_: Lin; Y_9_: Linolenc; Y_11_: Unsat; X_5_: SMC; X_6_: Soil moisture holding capacity ; X_7_: Soil saturated water content; X_8_: pH; X_9_: Soil total C content; X_10_: Soil total N content; X_12_: Soil total K content; X_14_: Soil total Mg content; X_15_: Soil total Ca content; X_17_: Soil total Mn content; X_18_: Soil total Fe content; X_20_: Soil total Zn content

## Discussion

### Soil physical and chemical properties affect ***C. oleifera*** root elements absorption

*C. oleifera* is an axial deep-rooted tree species, with 98.7% of its roots are concentrated in the 0-40 cm deep soil layer [25]. The physical and chemical properties of soil directly affect the absorption of plant nutrient elements [26]. *C. oleifera* root total N and P absorption were significantly dependent on the 0 ~ 20 cm soil, results similar to those reported by Li et al. [27]. As the surface layer of *C. oleifera* forest floor contain substantial amount of litter, it is expected that N and P are released after litter decomposition [28]. The more total N and P are released, the more conducive they are absorbed in the 20 ~ 40 cm soil layer [29]. In the 0 ~ 40 cm soil layer, pH value and total K content increased with soil depth, which are consistent with Cai et al., findings [30]. Root K absorption were significantly dependent on the 20 ~ 40 cm soil, and significantly and positively correlated with pH value. The observed increase in pH could be associated with increasing soil cation exchange capacity [31], and K element was adsorbed from the soil solution to soil particles and root surface, thus reducing leaching loss and increasing root K absorption [32]. *C. oleifera* root Mg, Fe, Cu and Ca absorption was significantly dependent on the total amount of these elements in soil layer, increased with the depth of the soil, and were significantly positively affected by SMC except for Cu, which consistent with those reported by Cao et al. [2]. So, increasing SMC, on the one hand, promoted the release of available Mg, Fe and Ca in the soil, thus improving root uptake [33, 34]; on the other hand, this promoted Cu soil fixation, leading to Cu absorption reduction [35]. *C. oleifera* root absorption of Al and Zn elements were not affected by the total amount of these elements in the soil. When the SMC decrease, the observed pH increase may reduce Mn, Al and Zn elements exchange capacity in soil [36], leading to reduced leaching loss and correspondingly increased Mn, Al and Zn elements root uptake. In conclusion, Fujian soil of *C. oleifera* forest was mainly regulated by SMC, pH, and total soil elements content.

At the same time, roots had synergistic and antagonistic effects during elements absorption [37]. The results indicated that when in humid soil, the pH value decrease, iron oxide film is formed on the root surface [38], increasing soil aqueous solution cation, releasing more soil phosphate, nitrate and ammonia, promoting *C. oleifera* N and P root absorption [39]. *C. oleifera* N absorption promoted root Ca absorption [40], and P absorption promoted Mg root absorption [41]. The iron oxide film adsorbed soil Al, Cu, Mn and Zn elements and reduced these elements root absorption [42]. Root ammonia and Ca absorption, and Cu fixation on the root surface inhibited root K absorption [43]. At the same time, phosphate fixing Al formed aluminum phosphate that was difficult for trees to utilize [44], which inhibited root Al absorption, which is similar to those reported by Qu et al. [45]. In conclusion, SMC, total N, P and Fe contents were the key factors controlling *C. oleifera* nutrient elements absorption in iron-rich soil.

### Transport rules of key nutrient elements affecting ***C. oleifera*** yield traits

Kernel P, N, Cu, Mn and K contents had the main effect on *C. oleifera* yield and fatty acid components, with P > Mn > Cu > N > K. Compared with other organs, kernel C, N, Mg, P and K contents were the largest above 1.50 g·Kg^− 1^, with C > K > N > P > Mg, which was similar to the results of Cao et al. [46]. It may be that K element enhancing the transport of carbohydrates to kernel [47], Mn and Cu elements activating growth enzymes activity [48], and P and N elements promoting protein synthesis [49], which were conducive to *C. oleifera* seed biomass growth. While P element as a component of phospholipid [50], Mg element promoting acetyl-CoA synthesis [51], which can accelerate fatty acids synthesis in *C. oleifera* kernel. It can be concluded that C, K, N, P, Mn and Cu elements may play a major role in the kernel growth and accumulation, while P, N and Mg elements may play a major role in kernel Linolenc, OCF, Ole, KOC, Unsat formation. These results indicated that the P, Mn, Cu, N, K, C and Mg elements determined *C. oleifera* kernel growth and development, which was similar to those reported by Cao et al. [52].

With the exception of C, the remaining P, N, Cu, Mn, K and Mg elements were absorbed from the soil by *C. oleifera* roots. SMC was conducive to *C. oleifera* root P element absorption. When the root P element is transported to leaves, leaves P content and had the largest positive effects with root N content, and negative effects with root Mn content. These indicated P element had synergistic and antagonistic effects with N and Mn elements, respectively. It is possible that during the transport of element P from root to leaves, N is favorable for P to form inorganic phosphorus, which is mainly transported upward with transpiration flow in ducts [53], and at the same time can exchange with Mn in the surrounding ductal cells and be unloaded into vacuoles of companion cells [54]. kernels P contents were positively greatest influenced by leaves Zn content, indicating that leaves P element had synergistic effect with Zn element transporting to kernel. It is possible that Zn element is a component of indoleacetic acid, and the fruit is the growth center after the Zn elements transfer from leaves to kernel, which promotes P transport [55]. So, root P content was determined by SMC, leaves P content was promoted by root N content and inhibited by root Mn content, kernel P content was promoted by leaves Zn content.

Root N content that positively and significantly influenced by SMC, had the greatest positive effect on leaves N content, thus positively effecting kernel N content. This indicate that moist soil was beneficial to *C. oleifera* root N absorption, mainly NO_3_^−^ part of which is absorbed by parenchyma cells around the ducts during transporting through xylem ducts, resulting in a decreasing trend of NO_3_^−^ concentration in xylem from root, stem, leaves to kernel [56]. So, root N content was determined by SMC, and the N content in other organs was determined by N content in the upstream organ from root to kernel.

Root K positively and significantly influenced by soil pH value, and root N content had the greatest negative effect on leaves K content. Leaves N content had the greatest negative effect on kernel K content, indicating that the increase of soil pH promoted K element absorption in *C. oleifera* roots, and the K content in organ was constrained by the N content in its upstream organ. It may be that K^+^ is transported in the xylem of plants, which competes for the ion channel with the transport of NO_3_ [56, 57]. Leaves Al content also had the greatest negative effect on kernel K content, and was enriched in *C. oleifera* leaves. It is possible that Al is a blocker of cation channels on the cell membrane, affects kernel K absorption of mineral elements by changing leaves plasma membrane fluidity and structure [58]. So, root K was determined by soil pH value, leaves K were inhibited by root N content, and kernel K were inhibited by leaves N and Al contents.

Root Cu and Mn contents positively and significantly influenced by the 20 ~ 40 cm soil layer pH value. Root K content was positive and significantly correlated with leaves Cu and Mn contents. These results indicated that the increase of soil pH promoted the uptake of Cu and Mn elements by *C. oleifera* roots, which transported from root to leaves through xylem and produces the “Viers effect” with K strongly influencing by transpiration [57]. Leaves N content had the greatest negative effect on kernel Cu and Mn contents, which are possible that N, Cu and Mn are involved in protein synthesis, reducing the transfer of Cu and Mn from leaves to the kernel [59]. So, root Cu and Mn determined by soil pH value, leaves Cu and Mn were promoted by root K content, and kernel Cu and Mn were inhibited by leaves N contents.

Root Mg content positively and significantly influenced by SMC in 20 ~ 40 cm soil layer. Root N content had the largest positive effect on leaves Mg content. The results indicated that the moist soil was conducive to Mg element absorption of *C. oleifera* root, When Mg element was transported from root to leaves, chlorophyll content is increased and nitrogen utilization in leaves is promoted, which has a synergistic effect with N [60]. kernel Mg content positively and significantly influenced by leaves Zn content, indicating Mg element was transported from leaves to kernel, has a synergistic effect with Zn. Mg participating in the phosphorylation process to promote substance synthesis, and Zn promoting the synthesis of indoleacetic acid, so Mg and Zn jointly promoting the growth of *C. oleifera* fruit [55, 60]. So, root Mg determined by SMC, leaves Mg were promoted by root N content, and kernel Mg were promoted by leaves Zn content.

In conclusion, in the soil of Fujian *C. oleifera* forest, increasing SMC was beneficial to the root absorption of P, N and Mg element; the proper increase of pH was beneficial to the root absorption of K, Cu and Mn element. Root N, P and Mg elements being promoted by root N element, root K element inhibiting by root N element, root P element inhibiting by root Mn element, root K element being promoted by root Cu and Mn element, transported from root to leaves. Leaves N element being promoted by leaves N element, leaves K, Cu and Mn element being inhibited by leaves N element, leaves P and Mg element being promoted by leaves Zn element, transported from leaves to kernel.

### Nutrient elements regulating ***C. oleifera*** oil yield

Plants absorb nutrient element in two ways, one by the soil and the other by the leaves [61]. The results showed that the capillary capacity in 0 ~ 20 cm soil layer had the most significant negative effects on OP, linoleic and Unsat. The soil saturated water content in 20 ~ 40 cm soil layer had the largest positive effect on OP and Ste, and had the largest negative effect on fruit yield, which indicated that soil moisture is the key factor of *C. oleifera* soil in the physical properties of soil and regulating *C. oleifera* yield and quality, which is similar to previous studies [62]. It may be related to the balance of soil moisture, air and nutrients [63]. The increase of capillary capacity in 0 ~ 20 cm soil layer will reduce soil aeration, which is not conducive to fatty acid synthesis [64]. The soil saturated water content of 20 ~ 40 cm soil layer increased, the soil element available content decreased, decreasing *C. oleifera* biomass and fruit yield. On the other hand, the more soil moisture, the higher the moisture content of kernel and pericarp, which is conducive to the transport of substances to *C. oleifera* fruit and the improvement of fatty acid synthesis [64, 65]. It can be concluded that the soil of *C. oleifera* forest should maintain moderate moisture content, which can not only improve the yield, but also improve the oil quality. Total Zn content in 0 ~ 20 cm soil layer which increases Ole, Lin, Linolenc and Unsat of *C. oleifera*, had the most significant positive effect on OP. It may be that Zn is an essential element for the synthesis of tryptophan [66], which is a precursor for the synthesis of indole acetic acid (IAA) and promotes the transport of assimilates to fruits growth [55]. Meanwhile, Zn is a cofactor of key glycolysis enzymes, which promotes the activity of aldoxase in plant tissues and promotes the synthesis of fats and unsaturated fatty acids [67]. The total Mn content in the 20 ~ 40 cm soil layer has the largest negative effect on the OP, which is different from previous studies that the element Mn can increase the OP [52]. It may be because the total Mn content in the 20 ~ 40 cm layer of three type soils were more than three times of the average of soil total Mn content [18], increases the Mn absorption, inhibits the enzyme synthesis [59], and reduce *C. oleifera* oil content. The total Mg content in the 20 ~ 40 cm soil layer had the largest significant positive effect on the fruit yield, and the total Ca content in the 20 ~ 40 cm soil layer had the largest significant positive effect on KOC which may be because magnesium improved the photosynthesis and promoted the transport of carbohydrates to fruits [60], thus increasing the fruit weight and the fruit yield. And Ca is the main component of oil drop, improving the conversion rate of oil [68]. In conclusion, the OP of *C. oleifera* could be improved by keeping proper SMC, applying Zn fertilizer in surface layer and Mg and Ca fertilizer in deep gully, and Ole, and Lin, Linolenc and Unsat be increased by applying Zn fertilizer in surface layer.

FWP was highly significant and positively correlated with leaves C, Mg and Zn contents, and was significant and positively correlated with OP, Unsat and KOC. It may be that Mg and C in leaves participate in photosynthesis [60] and Zn promotes the synthesis of indoleacetic acid [55], which jointly promotes the synthesis of energy and substance, and provides pericarp growth with storing energy and substance, then increases the OP and Unsat [69]. At the same time, the OP, Unsat and KOC were positively affected by kernel P content, which are similar to those reported by Cao et al. [2]. It is possible that element P promoted the first step reaction of Kennedy pathway [53], increasing *C. Oleifera* OP. Therefore, increasing the content of C, Mg and Zn in leaves and kernel P content can increase *C. Oleifera* OP, Unsat and KOC. KMC was highly significant and positive correlation with leaves K, Mn and Cu contents, significantly and positively related to Pal and Sat, significantly and negatively to OP, and Pal and Sat were highly significant and positive correlation with kernel K, Mn and Cu contents. It is possible that the contents of K, Mn and Cu in the leaves can increase the contents of K, Mn and Cu in the kernel. On the one hand, the vacuolar ion concentration can be increased, and increase the water content to reduce the osmotic pressure of the kernel [70]. On the other hand, cationic transport increased the substance content of kernel, and K, Mn and Cu could promote protein synthesis with providing sufficient fatty acid synthase [57, 59]. Therefore, increasing the contents of K, Mn and Cu in leaves can increase the contents of Pal and Sat, but decrease of OP, similar to those previously reported by Cao et al. [52]. FSI and leaves N, Al and Cu contents, KFW and leaves P, N and Mg contents were significant or highly significantly and positively correlated, FSI and KFW were highly significant and positive correlation with OCF, Linolenc, Ole and Ste, KFW were highly significant and positive correlation, FSI were highly significant and negative with OP. These were indicating that moderate leaves N content, decreasing leaves Al and Cu, and increasing leaves P and Mg contents could increase OP, and OCF, Linolenc, Ole and Ste. In conclusion, by observing FWP, KMC, FSI and KFW, leaves N, P, Mg, Zn content can be increase through applying N, P, Mg, Zn leaf fertilization to affect the fruit development, and then the OP and Unsat of *C. oleifera* can be increased.

## Conclusions

**Fig. 6:**
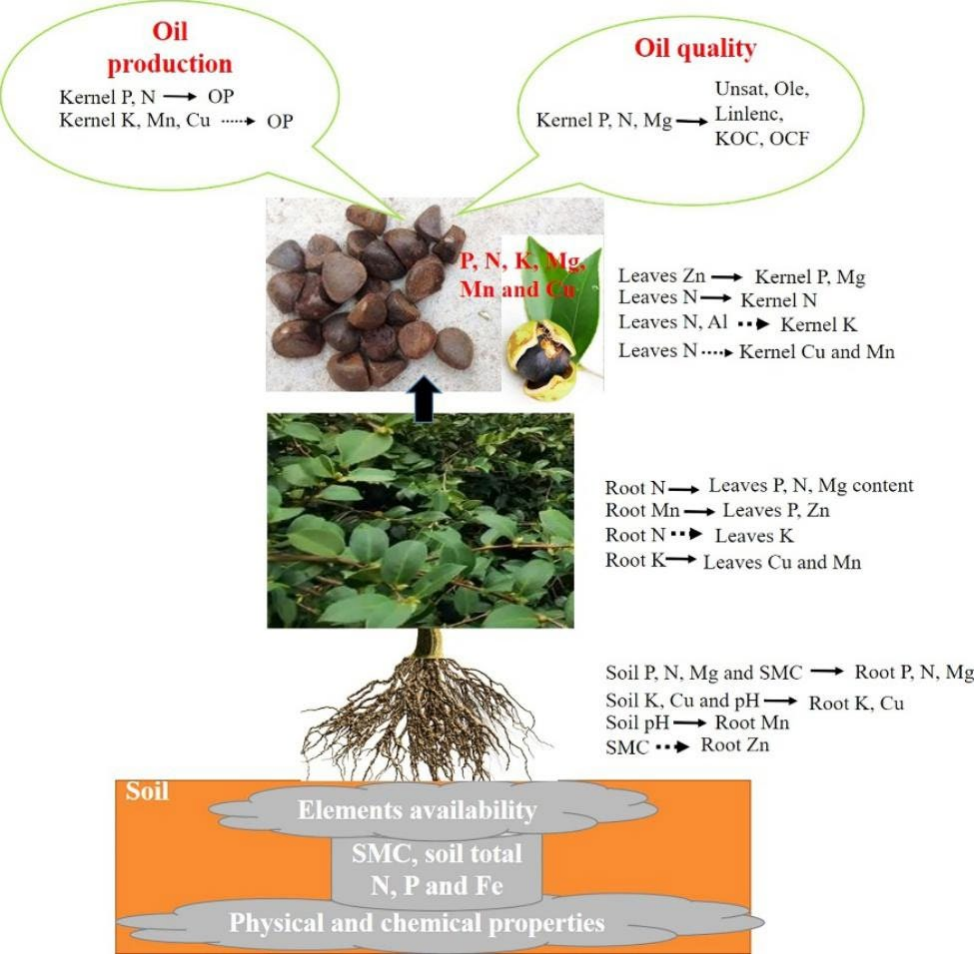
Role of soil nutrient transport on *C. oleifera* yield and quality under different soil types

Available elements contents in the soil of *C. oleifera* plantations in Fujian were mainly regulated by SMC, soil pH and total soil element content. Soil total N, P, K, Mg, Fe, Cu and Ca significantly affected the root absorption, while soil total Mn, Al and Zn had no correlation with the root absorption, in which soil total N, P and Fe content were the key points to control nutrient elements absorption of *C. oleifera*. N, P, K, Mg, Cu, Mn and C element were the key elements for the kernel growth and the yield traits of *C. oleifera*. Root P, N and Mg contents were determined by SMC; root K, Cu and Mn contents were determined by soil pH value. Leaves N, P and Mg contents were promoted by root N content; leaves K and P was inhibited by root N and Mn content, respectively; leaves Cu and Mn were promoted by root K content. Kernel P and Mg content were promoted by leaves Zn content; kernel N content was promoted by leaves N content; kernel K, Cu and Mn were inhibited by leaves N contents, and kernel K were inhibited by leaves Al contents. The OP and each oil component content can be regulated by two ways. The OP and Unsat of *C. oleifera* could be improved by keeping proper SMC, applying Zn fertilizer in surface layer and Mg and Ca fertilizer in deep gully. The other was to by observing FWP, KMC, FSI and KFW, through applying N, P, Mg, Zn leaf fertilization, could increase leaves N, P, Mg, Zn content to affect the fruit development, then the OP and Unsat of *C. oleifera* can be increased (Fig. 6).

## Materials and methods

### Overview of the study area and plot setting

The main soil types of *C. oleifera* in Fujian Province (China) are red, yellow-red, and purple soil. The formal identification of *C. oleifera* was undertaken by Dr. Wenjun Lin (Fujian Agriculture and Forestry University), and a voucher specimen of *C. oleifera* has been deposited in a public herbarium of Fujian Agriculture and Forestry University. In November 2020 (fruit ripening period), three typical mature *C. oleifera* forests with the same management practices, site conditions, and canopy density were selected in Minhou, Taining, and Ninghua counties, Fujian Province, representing red, yellow-red, and purple soil, respectively. *C. oleifera* forests are 12–15 years old and about 2.2 m tall. For each soil type, standard 20 m × 20 m plots were set with three replications per soil type. The study area has a mid-subtropical monsoon climate, and environmental factors in each sampling area were showed in Table 4.

**Table 4:** Environmental factors in each sampling area

Environmental factor	Minhou county	Taining county	Ninghua county
Longitude	N25°47′～26°36′	N26°34′～27°08′	N25°58′～26°40′
Latitude	E118°52′～119°25′	E116°53′～117°24′	E116°22′～117°02′
average annual temperature (℃)	14.8～19.5	15.0～18.0	15.0～18.0
average annual precipitation (mm)	1673.9	1725.0	1787.6

### Sample collection

Within each plot, five trees with medium growth and free from disease and insect were selected as the sampling plants. In the first ten days of November 2020, samples were taken at fruit ripening period of *C. oleifera*.

Soil samples. Under the drip line of each sample tree’s canopy, four cardinal directions (east, south, west and north) points were determined, and 0–20 cm and 20–40 cm ring-knife soil and scattered soil samples were collected, respectively [71]. From each layer (soil and scattered soil), 3 ring-knives samples were collected for soil physical properties determination. After mixing the same layer of scattered soil samples collected from each plot and replication, plant residues, gravel, and other debris were discarded. For each replication, about 1 kg of soil was taken by quartering method, and brought back to the laboratory for natural air drying, crushed and ground, passed through a 60-mesh nylon sieve, and stored in sealed bags for soil nutrient content and soil pH determination.

Plant samples. During soil sampling, the fine roots mixed in the soil were collected. From the middle canopy of each sampling tree, 1-year-old branches with moderate growth were selected, and the 2nd to 3rd leaves from the branches’ top were evenly collected from the four cardinal directions [72]. A total of about 20 leaves were collected from each tree. The roots and leaves collected from each sample were mixed and brought back to the laboratory, dried at 60 °C to a constant mass, crushed and ground, passed through a 60-mesh sieve, and stored in sealed bags for nutrient content determination. After counting the number of fruits of each sample tree, 20 fruits were randomly selected from the four cardinal directions, and brought to the laboratory for further use.

### Soil physical and chemical properties determination

A 0.5 g of dry soil sample passing through a 10-mesh sieve was used to determine the C and N contents using a Vario MACRO Cube carbon and nitrogen elemental analyzer (Elementar, Germany). Additional 0.5 g dry soil sample passing through a 10-mesh sieve, was placed in a digestion cup, wetted with deionized water, then 5 mL aqua rega (mixture of concentrated nitric and hydrochloric acids) and 3 ml perchloric acid were added, and placed in a preheated graphite digestion instrument. After digestion at 260 °C for 3 h, the soil emitted white smoke and turned gray, then the heating stopped. After cooling, excess perchloric acid was removed, and 5 mL of 2% nitric acid was added, heated at 140 °C for 3 min, cooled, filtered until clarification, added 2% nitric acid to make the volume to 50 mL, and stored in a digester bottle for later use. Total P, K, Ca, Mg, Cu, Fe, Al, Zn, and Mn contents were determined by Optima 8 000 ICP-OES inductively coupled plasma emission spectrometer (PerkinElmer, USA) [73]. Soil pH was measured by potentiometric method [74], soil moisture content (SMC), moisture holding capacity and saturated water content were measured by drying method [75], and soil bulk density, total soil porosity, capillary porosity, and non-capillary porosity were measured by ring-knife method [76].

### Plant organ elements determination

A 0.1 g of dry plant sample was used for C and N contents determination using a Vario MACRO Cube carbon and nitrogen element analyzer (Elementar, Germany). Furthermore, a 0.2 g dry plant sample was digested by microwave using Milestone Ethos Up Microwave Digestion instrument (Italian company Milestone). After digestion, access acid was removed at 160 ℃ for 3 ~ 4 h, fixed to 50 mL by the digestion solution, filtered by filter paper. Finally, total P, K, Ca, Mg, Cu, Fe, Al, Zn, and Mn contents were determined by Optima 8 000 ICP-OES inductively coupled plasma emission spectrometer (PerkinElmer, USA) [73].

### Fruit and yield traits determination

Using 20 fresh fruits from each sampling plant, fruits were measured for height (mm) and diameter (mm) with a vernier caliper, and fruit shape index (FSI) was calculated, as follows: FSI = fruit height / fruit diameter. Individual fruit fresh weight (g) was measured by an electronic scale, and the fruit yield (g·plant^− 1^) was calculated as follows: the fruit yield = the average fruit fresh weight × number of fruits per plant. After removing the seeds and pericarp from the fruit, the number of seeds per fruit was counted. Seed and fresh weight of pericarp (FWP) (g) were determined by electronic scale, and the fresh seed rate % = total weight of fresh seed / total weight of fresh fruit ×100%. Then the kernel and seed coat were removed from the seeds, and the kernel fresh weight (KFW) (g) was measured by electronic balance. The pericarp thickness (mm) at the fruit base, middle, and top were measured by vernier calipers. The peel, kernel, and seed coat were placed in a drying oven at 105 ℃ and dried at 60 ℃ to a constant mass. The dry weights of fruit, peel, seed, and kernel were determined as well as their moisture contents were calculated as follows: (a) fruit moisture contents % = (fruit fresh weight - dry weight of pericarp - kernel dry weight - dry weight of seed coat) / fruit fresh weight ×100%, (b) pericarp moisture content % = (fresh weight of pericarp - pericarp dry weight) / fresh weight of pericarpt×100%, (c) seed moisture content % = (seed fresh weight - kernel dry weight - seed coat dry weight) / seed fresh weight ×100%, (d) kernel moisture content % = (kernel fresh weight - kernel dry weight) / kernel fresh weight ×100%. After drying, the kernels were ground into powder by high-speed universal pulverizer (AISTTE Tianjin Tester Instrument Co., LTD.), part of which was screened by 60 mesh and stored in sealed bags for nutrient content determination and the other part was used for kernel oil extraction by Soxhlet extractor, after which the dried kernel oil content (KOC) was measured, and the crude oil was obtained [77]. Oil content of fresh fruit (OCF) % = kernel dry weight × dry kernel oil content / fresh fruit total weight, and oil production per plant (OP) (g·plant^− 1^) = fruit yield per plant × OCF. A sample of accurately weighed 0.5 g crude oil was used to determine palmitic acid content (Pal), stearic acid content (Ste), oleic acid content (Ole), linoleic acid content (Lin), linolenic acid content (Linolenc), and other composition content of each oil by gas chromatography-mass spectrometry [78]. These contents were determined as follow: (a) saturated fatty acid content (Sat) % = Pal + Ste; (b) unsaturated fatty acid content (Unsat) % = Ole + Lin + Linolenc; (c) saturated/unsaturated ratio (Sat/Unsat) = saturated fatty acid content / unsaturated fatty acid content [79] .

### Data analysis

Excel 2010 was used for data collation, and all data were described by mean and standard deviation (SD). DPS 19.05 software was used for statistical analysis, one-way ANOVA was used to analyze the differences of each index in different soil types, and double-screening stepwise regression was used to analyze the relationship between soil physical and chemical properties and yield traits to determine which soil physical and chemical properties impact which yield traits [17]. According to the significance test results of the variance contribution of each soil physical and chemical character, some soil physical and chemical characters that contributed more to the variance of a certain yield character were selected, and regression models were constructed according to the yield character [18]. Pearson correlation analysis was used to analyze the relationship between fruit characters and leaves element contents. Canoco 5.0 software was used for redundancy analysis (RDA) of soil, root, leaf, kernel, fruit and yield traits to determine the main influencing factors [80]. The explanatory variables with *P* < 0.05 were manually introduced to obtain a two-dimensional ranking map, which can visually reflect the relationship between explanatory variables and response variables (explanatory variables are represented by solid red lines with arrows, response variables are represented by solid blue lines with arrows, and the angles between all vectors reflect their linear correlations, which are equal to the cosine of the angles between vectors [80]. In this study, correlation coefficient greater than 0.487 indicated highly significant correlation between indicators, that is, in the two-dimensional ranking map, when the angle between vectors was < 60°, there was highly significant positive correlation between vectors, and when the angle was > 120°, there was highly significant negative correlation between vectors. The vertical projection length of the explanatory variable arrow segment on the response variable factor arrow segment is used to represent the degree of influence of the explanatory variable on the response variable. The longer the length, the greater the influence [81].

## Data Availability

The original contributions presented in the study are included in this article. Further inquiries can be directed to the corresponding author.

## Abbreviations

FSI: Fruit shape index
OCF: Oil content of fresh fruit
KMC: Kernel moisture content
SMC: Soil moisture content
FWP: Fresh weight of pericarp
pH: Soil pH
KFW: Kernel fresh weight
C: C content
Ste: Stearic acid content
N: N content
Sat: Saturated fatty acid content
P: P content
Unsat: Unsaturated fatty acid content
K: K content
Sat/ Unsat: Saturated/unsaturated ratio
Mg: Mg content
Pal: Palmitic acid content
Ca: Ca content
Lin: Linoleic acid content
Al: Al content
OP: Oil production
Mn: Mn content
KOC: Dry kernel oil content
Fe: Fe content
Ole: Oleic acid content
Cu: Cu content
Linolenc: Linolenic acid content
Zn: Zn content

## References

[1] Wen Y, Zhang Y, Su S, Yang S, Ma L, Zhang L (2019). Effects of tree shape on the microclimate and fruit quality parameters of *Camellia oleifera* Abel. Forests.

[2] Cao YQ, Yao XH, Ren HD, Wang KL, Li JX, Zhou Q (2022). Correlations between soil nutrient characteristics and kernel quality traits in *Camellia oleifera* cultivation. Chin Agric Sci Bull.

[3] Liu J, Wu L, Chen D, Yu Z, Wei C (2018). Development of a soil quality index for *Camellia oleifera* forestland yield under three different parent materials in Southern China. Soil Tillage Res.

[4] Dai Y, Chen F, Yue L, Li T, Jiang Z, Xu Z (2020). Uptake, transport, and transformation of CeO_2_ nanoparticles by strawberry and their impact on the rhizosphere bacterial community. ACS Sustain Chem Eng.

[5] Rietra RP, Heinen M, Dimkpa CO, Bindraban PS (2017). Effects of nutrient antagonism and synergism on yield and fertilizer use efficiency. Commun Soil Sci Plant Anal.

[6] Bornø ML, Müller-Stöver DS, Liu F (2019). Biochar properties and soil type drive the uptake of macro-and micronutrients in maize (*Zea mays* L). J Plant Nutr Soil Sci.

[7] Alaoui I, el ghadraoui O, Serbouti S, Ahmed H, Mansouri I, El Kamari F (2022). The mechanisms of absorption and nutrients transport in plants: a review. Trop J Nat Prod Res.

[8] Xu T, Cui K, Chen J, Wang R, Wang X, Chen L (2021). Biodiversity of culturable endophytic actinobacteria isolated from high yield *Camellia oleifera* and their plant growth promotion potential. Agriculture.

[9] Latifinia E, Eisvand HR (2022). Soybean physiological properties and grain quality responses to nutrients, and predicting nutrient deficiency using chlorophyll fluorescence. J Soil Sci Plant Nutr.

[10] Muhammad I, Lv JZ, Yang L, Ahmad S, Farooq S, Zeeshan M (2022). Low irrigation water minimizes the nitrate nitrogen losses without compromising the soil fertility, enzymatic activities and maize growth. BMC Plant Biol.

[11] Tu J, Chen J, Zhou J, Ai W, Chen L (2019). Plantation quality assessment of *Camellia oleifera* in mid-subtropical China. Soil Tillage Res.

[12] Liu L, Zhang L, Pan J, Niu J, Yuan X, Hu S (2020). Soil CNP pools and stoichiometry as affected by intensive management of *Camellia oleifera* plantations. PLoS ONE.

[13] Zhang Z, Tariq A, Zeng F, Graciano C, Zhang B (2020). Nitrogen application mitigates drought-induced metabolic changes in *Alhagi sparsifolia* seedlings by regulating nutrient and biomass allocation patterns. Plant Physiol Biochem.

[14] Milošević T, Milošević N, Mladenović J (2022). The influence of organic, organo-mineral and mineral fertilizers on tree growth, yielding, fruit quality and leaf nutrient composition of apple cv. ‘Golden Delic Reinders’ Scientia Horticulturae.

[15] Luo J, Wei S, Zhou X, Tian Y, Chen Y, Song Q (2022). Nutrient contents in the organs and soil of young and mature *Camellia oleifera* C. Abel forests in China. Bangladesh J Bot.

[16] Kome GK, Enang RK, Tabi FO, Yerima BPK (2019). Influence of clay minerals on some soil fertility attributes: a review. Open J Soil Sci.

[17] Tian ZK, Yang FH, Yin C (2014). Many-to-many regression analysis of loading characteristic in dieless tube hydroforming. Appl Mech Mater.

[18] Liu SQ, Bian Z, An TY, Xia CZ, Zhang M, Chen J (2021). Carbon pools of biomass and dead organic matter in typical forest ecosystems of Tibet: a new estimation based on the first forestry carbon sequestration monitoring undertaken in China. Land Degrad Dev.

[19] Thorne ED, Ford WM (2022). Redundancy analysis reveals complex den use patterns by eastern spotted skunks, a conditional specialist. Ecosphere.

[20] Zhang DM, Xie LS, Zhang W, Zheng DJ, Pan XZ, Zeng JH (2015). Investigation and evaluation on soil fertility of main *Camellia oleifera* forests in Hainan. Nonwood For Res.

[21] Yan B, Hou Y. Effect of soil magnesium on plants: a review//IOP conference series: earth and environmental science. IOP Publishing. 2018;170:022168.

[22] Zhu Y, Weindorf DC (2009). Determination of soil calcium using field portable x-ray fluorescence. Soil Sci.

[23] Guo J, Xuan F, Li D, Wang J, Zhang B (2022). Variations of soil chemical properties and microbial community around the acid reservoir in the mining area. Sustainability.

[24] Bhatti SS, Sambyal V, Nagpal AK (2018). Analysis of genotoxicity of agricultural soils and metal (Fe, Mn, and zn) accumulation in crops. Int J Environ Res.

[25] Yang J, Tan XF, Yuan DY, Peng X, Jiang YL (2009). Study on root system distribution of *Camellia oleifera*. J Zhejiang Forestry Sci Technol.

[26] Yin X, Zhao L, Fang Q, Ding G (2021). Differences in soil physicochemical properties in different-aged *Pinus massoniana* plantations in Southwest China. Forests.

[27] Li RQ, Xu YL, Zhu W, Jiao JF, Xi RC (2015). Biomass and nutrient distribution of *Camellia oleifera*. Guangdong Agric Sci.

[28] Zheng Y, Guan F, Fan S, Yan X, Huang L (2022). Dynamics of leaf-litter biomass, nutrient resorption efficiency and decomposition in a moso bamboo forest after strip clearcutting. Front Plant Sci.

[29] Chen X, Yan X, Wang M, Cai Y, Weng X, Su D (2022). Long-term excessive phosphorus fertilization alters soil phosphorus fractions in the acidic soil of pomelo orchards. Soil Tillage Res.

[30] Cai PJ, Cao SJ, Chen AL, Huang TS, Cao GQ (2017). Variation analysis of soil physical and chemical properties of different forest types from purple soil areas in Ninghua County. Subtropical Agric Res.

[31] Zhou T, Zhao M, Zhao X, Guo Y, Zhao Y (2021). Simultaneous remediation and fertility improvement of heavy metals contaminated soil by a novel composite hydrogel synthesized from food waste. Chemosphere.

[32] Xiao Y, Chen L (2022). Arbuscular mycorrhizal fungi reduce potassium, cadmium and ammonium losses but increases nitrate loss under high intensity leaching events. BMC Plant Biol.

[33] Huang Y, Wang M, Li Z, Gong Y, Zeng EY (2019). In situ remediation of mercury-contaminated soil using thiol-functionalized graphene oxide/Fe-Mn composite. J Hazard Mater.

[34] He K, He G, Wang C, Zhang H, Xu Y, Wang S (2020). Biochar amendment ameliorates soil properties and promotes Miscanthus growth in a coastal saline-alkali soil. Appl Soil Ecol.

[35] Li S, Sun X, Li S, Liu Y, Ma Q, Zhou W (2021). Effects of amendments on the bioavailability, transformation and accumulation of heavy metals by pakchoi cabbage in a multi-element contaminated soil. RSC Adv.

[36] Hailegnaw NS, Mercl F, Pračke K, Praus L, Száková J, Tlustoš P (2020). The role of biochar and soil properties in determining the available content of Al, Cu, Zn, Mn, and cd in soil. Agronomy.

[37] Xie K, Cakmak I, Wang S, Zhang F, Guo S (2021). Synergistic and antagonistic interactions between potassium and magnesium in higher plants. Crop J.

[38] Zhou S, Liu Z, Sun G, Zhang Q, Cao M, Tu S (2022). Simultaneous reduction in cadmium and arsenic accumulation in rice (*Oryza sativa* L.) by iron/iron-manganese modified sepiolite. Sci Total Environ.

[39] Preetha PS, Balakrishnan N (2017). A review of nano fertilizers and their use and functions in soil. Int J Curr Microbiol Appl Sci.

[40] Xing Y, Zhu ZL, Wang F, Zhang X, Li BY, Liu ZX (2021). Role of calcium as a possible regulator of growth and nitrate nitrogen metabolism in apple dwarf rootstock seedlings. Sci Hort.

[41] Faria JM, Teixeira DM, Pinto AP, Brito I, Barrulas P, Alho L (2020). Toxic levels of manganese in an acidic cambisol alters antioxidant enzymes activity, element uptake and subcellular distribution in *Triticum aestivum*. Ecotoxicol Environ Saf.

[42] Suda A, Makino T (2016). Functional effects of manganese and iron oxides on the dynamics of trace elements in soils with a special focus on arsenic and cadmium: a review. Geoderma.

[43] Sustr M, Soukup A, Tylova E (2019). Potassium in root growth and development. Plants.

[44] Hii YS, San CY, Lau SW, Danquah MK (2020). Isolation and characterisation of phosphate solubilizing microorganisms from peat. Biocatal Agric Biotechnol.

[45] Qu XJ, Chen M, Liao J, Zhang CH, Chen LS, Yuan J (2021). Physiological response of phosphorus alleviates aluminum stress in leaves of *Camellia oleifera*. J Cent South Univ Forestry Technol.

[46] Cao YQ, Ren HD, Lin P, Wang KL, Yao XH, Long W (2012). Research on annual changes of nitrogen, phosphorous, potassium absorption and accumulation in oil-tea camellia tree. For Res.

[47] Luo A, Kang S, Chen J (2020). SUGAR model-assisted analysis of carbon allocation and transformation in tomato fruit under different water along with potassium conditions. Front Plant Sci.

[48] Türkan F, Atalar MN, Aras A, Gülçin İ, Bursal E (2020). ICP-MS and HPLC analyses, enzyme inhibition and antioxidant potential of *Achillea schischkinii* Sosn. Bioorg Chem.

[49] Chaieb N, Rezguia M, Ayedb S, Bahria H, Cheikh H, M’hameda MR (2020). Effects of tillage and crop rotation on yield and quality parameters of durum wheat in Tunisia. J Anim Plant Sci.

[50] Lim HY, Wang W, Wessells RJ, Ocorr K, Bodmer R (2011). Phospholipid homeostasis regulates lipid metabolism and cardiac function through SREBP signaling in Drosophila. Genes Dev.

[51] Adina-Zada A, Zeczycki TN, Attwood PV (2012). Regulation of the structure and activity of pyruvate carboxylase by acetyl CoA. Arch Biochem Biophys.

[52] Cao YQ, Yao XH, Ren HD, Wang KL (2015). Changes in contents of endogenous hormones and main mineral elements in oil-tea *Camellia* fruit during maturation. J Beijing Forestry Universty.

[53] Nehls U, Plassard C (2018). Nitrogen and phosphate metabolism in ectomycorrhizas. New Phytol.

[54] Pongrac P, Baltrenaite E, Vavpetič P, Kelemen M, Kladnik A, Budič B (2019). Tissue-specific element profiles in Scots pine (*Pinus sylvestris* L.) needles. Trees.

[55] Kang SM, Shahzad R, Khan MA, Hasnain Z, Lee KE, Park HS (2021). Ameliorative effect of indole-3-acetic acid-and siderophore-producing *Leclercia adecarboxylata* MO1 on cucumber plants under zinc stress. J Plant Interact.

[56] Elejoste A, Arevalillo A, Gabilondo N, Butron A, Peña-Rodriguez C (2021). Morphological analysis of several bamboo species with potential structural applications. Polymers.

[57] Panda BB, Sharma SG, Mohapatra PK, Das A (2012). Iron stress induces primary and secondary micronutrient stresses in high yielding tropical rice. J Plant Nutr.

[58] Liu H, Zhu R, Shu K, Lv W, Wang S, Wang C (2022). Aluminum stress signaling, response, and adaptive mechanisms in plants. Plant Signal Behav.

[59] Hänsch R, Mendel RR (2009). Physiological functions of mineral micronutrients (Cu, Zn, Mn, Fe, Ni, Mo, B, cl). Curr Opin Plant Biol.

[60] Pranckietiene I, Dromantiene R, Jodaugiene D, Vaguseviciene I, Paulauskiene A, Marks M (2020). Effect of liquid amide nitrogen fertilizer with magnesium and sulphur on spring wheat chlorophyll content, accumulation of nitrogen and yield. J Elementology.

[61] He C, Zhang L, Li X (2022). Plant performance and soil fungal community impacts of enhancing *Dioscorea opposit* a with spraying foliar fertilizer with different nutrient element combinations[J]. Agronomy.

[62] Zhong FX, Wang RH, Li T, Zhou P, Liao WT, Zhou YG (2015). Effects of soil moisture on major economic indexes of *Camellia oleifera* fruits. Nonwood For Res.

[63] Tahoun AMMA, El-Enin MMA, Mancy AG, Sheta MH, Shaaban A (2022). Integrative soil application of humic acid and foliar plant growth stimulants improves soil properties and wheat yield and quality in nutrient-poor sandy soil of a semiarid region. J Soil Sci Plant Nutr.

[64] Ben-Noah I, Friedman SP (2018). Review and evaluation of root respiration and of natural and agricultural processes of soil aeration. Vadose Zone J.

[65] Plett DC, Ranathunge K, Melino VJ, Kuya N, Uga Y, Kronzucker HJ (2020). The intersection of nitrogen nutrition and water use in plants: new paths toward improved crop productivity. J Exp Bot.

[66] Sourati R, Sharifi P, Poorghasemi M, Alves Vieira E, Seidavi A, Anjum NA (2022). Effects of naphthaleneacetic acid, indole-3-butyric acid and zinc sulfate on the rooting and growth of mulberry cuttings. Int J Plant Biology.

[67] Wassie T, Duan X, Xie C, Wang R, Wu X (2022). Dietary Enteromorpha polysaccharide-Zn supplementation regulates amino acid and fatty acid metabolism by improving the antioxidant activity in chicken. J Anim Sci Biotechnol.

[68] Zhao W, Liu J, Qian L, Guan M, Guan C (2022). Genome-wide identification and characterization of oil-body-membrane proteins in polyploid crop *Brassica napus*. Plants.

[69] Wang C, Qi M, Guo J, Zhou C, Yan X, Ruan R (2022). The active phytohormone in microalgae: the characteristics, efficient detection, and their adversity resistance applications. Molecules.

[70] Lethin J, Byrt C, Berger B, Brien C, Jewell N, Roy S (2022). Improved salinity tolerance-associated variables observed in EMS mutagenized wheat lines. Int J Mol Sci.

[71] Sileshi GW (2016). The magnitude and spatial extent of influence of *Faidherbia albida* trees on soil properties and primary productivity in drylands. J Arid Environ.

[72] Chen Y, Li Y, Wu M, Lu F, Hou M, Yin Y (2022). Differentiating Crohn’s disease from intestinal tuberculosis using a fusion correlation neural network. Knowl Based Syst.

[73] Myrvang MB, Hillersøy MH, Heim M, Bleken MA, Gjengedal E (2016). Uptake of macro nutrients, barium, and strontium by vegetation from mineral soils on carbonatite and pyroxenite bedrock at the Lillebukt Alkaline Complex on Stjernøy, Northern Norway. J Plant Nutr Soil Sci.

[74] Błońska E, Lasota J (2017). Soil organic matter accumulation and carbon fractions along a moisture gradient of forest soils. Forests.

[75] Li S, Bowker MA, Chamizo S, Xiao B (2022). Effects of moss biocrusts on near-surface soil moisture are underestimated in drylands: insights from a heat-pulse soil moisture sensor. Geoderma.

[76] Zhu J, Cao Y, He W, Xu Q, Xu C, Zhang X (2021). Leaf functional traits differentiation in relation to covering materials of urban tree pits. BMC Plant Biol.

[77] Wu H, Li C, Li Z, Liu R, Zhang A, Xiao Z (2018). Simultaneous extraction of oil and tea saponin from *Camellia oleifera* Abel. Seeds under subcritical water conditions. Fuel Process Technol.

[78] Shi T, Wu G, Jin Q, Wang X (2022). *Camellia* oil adulteration detection using fatty acid ratios and tocopherol compositions with chemometrics. Food Control.

[79] Tao L, Qin W, Wei Z, Li X, Zhang H (2022). Effects of small-scale storage on the cooking property and fatty acid profile of sea rice paddy. Appl Food Res.

[80] Misztal M (2017). On the Use of redundancy analysis to study the property crime in Poland. Acta Universitatis Lodziensis Folia Oeconomica.

[81] Garibay MV, Castillo AF, Torres OD, Anda J, Yebra-Montes C, Senés-Guerrero C (2021). Characterization of the spatial variation of microbial communities in a decentralized subtropical wastewater treatment plant using passive methods. Water.

